# Infectivity-Enhanced, Conditionally Replicative Adenovirus for COX-2-Expressing Castration-Resistant Prostate Cancer

**DOI:** 10.3390/v15040901

**Published:** 2023-03-31

**Authors:** Tatyana Gavrikova, Naohiko Nakamura, Julia Davydova, Emmanuel S. Antonarakis, Masato Yamamoto

**Affiliations:** 1 Division of Human Gene Therapy, Department of Surgery, Medicine and Pathology, University of Alabama at Birmingham, Birmingham, AL 35294, USA; 2 Division of Basic and Translational Research, Department of Surgery, University of Minnesota, Minneapolis, MN 55455, USA; 3 Masonic Cancer Center, University of Minnesota, Minneapolis, MN 55455, USA; 4 Division of Hematology, Oncology and Transplantation, Department of Medicine, University of Minnesota, Minneapolis, MN 55455, USA

**Keywords:** neuroendocrinal prostate cancer, NEPC, prostate cancer, cyclooxygenase, virotherapy, castration resistant, adenoviral, replicative

## Abstract

Background: The development of conditionally replicative adenoviruses (CRAds) for castration-resistant prostate cancer (CRPC), particularly neuroendocrine prostate cancer (NEPC), has two major obstacles: choice of control element and poor infectivity. We applied fiber-modification-based infectivity enhancement and an androgen-independent promoter (cyclooxynegase-2, COX-2) to overcome these issues. Methods: The properties of the COX-2 promoter and the effect of fiber modification were tested in two CRPC cell lines (Du-145 and PC3). Fiber-modified COX-2 CRAds were tested in vitro for cytocidal effect as well as in vivo for antitumor effect with subcutaneous CRPC xenografts. Results: In both CRPC cell lines, the COX-2 promoter showed high activity, and Ad5/Ad3 fiber modification significantly enhanced adenoviral infectivity. COX-2 CRAds showed a potent cytocidal effect in CRPC cells with remarkable augmentation by fiber modification. In vivo, COX-2 CRAds showed an antitumor effect in Du-145 while only Ad5/Ad3 CRAd showed the strongest antitumor effect in PC3. Conclusion: COX-2 promoter–based, infectivity-enhanced CRAds showed a potent antitumor effect in CRPC/NEPC cells.

## 1. Introduction

Prostate cancer (PC) is a major public health problem across the globe. With >1 million new cases and >300,000 deaths annually, prostate cancer is the second most common cancer and the fifth leading cause of cancer death in men worldwide [1]. Even after the widespread adoption of prostate-specific antigen (PSA) screening, a considerable number of patients are still found with locally advanced or metastatic diseases [2]. Androgen-deprivation therapy (ADT) has been the cornerstone for advanced and metastatic PC. However, despite initial high response rates, management with ADT is effective only for a limited duration; nearly all men eventually develop progressive prostate cancer following ADT, causing castration-resistant prostate cancer (CRPC) [3]. Most PC patients with metastasis develop CRPC within 5 years of follow up, and the median survival after the development of castration resistance is approximately 2–3 years [4]. In addition, 15–33% patients with nonmetastatic CRPC can progress to metastatic disease within 2 years, increasing the mortality burden in this population [5]. Therefore, it is essential to prevent metastatic CRPC (mCRPC) development and establish a new therapeutic strategy for mCRPC. Currently, there are multiple approved systemic therapies able to prolong the survival of patients with mCPRC [6]. Although these therapies each improved overall survival by three–four months, they are only temporarily effective, and the overall survival of patients with mCRPC still remains poor [7]. To improve the prognosis of patients with advanced PC, more specific, targeted therapy for mCRPC is urgently required.

A particularly challenging problem is the management of AR-indifferent prostate cancers that either do not express AR from the onset of the cancer or lose AR expression during the course of systemic therapy. The former are typically designated as small-cell prostate cancers, while the latter are termed treatment-related neuroendocrine prostate cancers (t-NEPC). Among CRPCs, patients developing treatment-related NEPC (t-NEPC) show significant resistance against standard chemotherapy regimens and all hormonal regimens [8,9]. The National Comprehensive Cancer Network (NCCN) guidelines therefore include consideration of metastatic biopsy in any patient with CRPC to look for t-NEPC transformation, since this will change management if found [10]. To recapitulate small-cell prostate cancer and t-NEPC states both represent unmet medical needs, we chose to utilize the PC3 and Du-145 cell lines (PMID: 14518029) in this study. PC3 is an AR-negative small-cell prostate cancer cell line derived from a metastatic bone lesion. Du-145 is an AR-negative t-NEPC cell line derived from a dural brain metastasis. Both cell lines are notoriously difficult to treat in preclinical settings [8,11,12].

Cyclooxygenase-2 (COX-2), which is primarily responsible for prostaglandins produced in inflammatory sites, is reported to be upregulated in various cancer tissues and plays an important role in the disease progression of breast, colon, lung, and bladder cancers, as well as in prostate cancer [13,14,15,16,17]. COX-2 is a well-known factor that affects malignant aggressiveness and worsens the prognosis in PC patients. Previous clinical study indicated that increased COX-2 expression in PC tissues was significantly associated with worse therapeutic outcomes and distant metastasis development [18]. In addition, COX-2 plays important roles in the malignant behavior of CRPC [19]. In fact, CRPC tissues showed significantly higher expression of COX-2 than hormone-naïve PC tissues [20], and the inhibition of the COX-2 pathway downregulates a number of important targets of CRPC including androgen receptors [21]. Interestingly, in the PC3 cell line, it was reported that the COX-2 inhibitor induced significant tumor regression in a xenograft model [22]. Hence, COX-2 offers a relevant therapeutic target in CRPC patients who already have metastasis or a higher metastatic risk. Eliminating COX-2-positive CRPC cells could contribute to the control of mCRPC and improve therapeutic outcomes and prognosis in CRPC patients.

Oncolytic virotherapy is a promising therapeutic approach employing the cytocidal function of viruses in order to kill cancer cells. In recent years, the development of oncolytic viruses and their implementation in clinical trials have gained increased attention [23]. Adenovirus (Ad) has been the most popular choice of vector in the clinical trials due to its high transduction efficiency in vivo as well as in vitro [24]. Human Ad has been used extensively to develop replication-deficient gene delivery vectors and conditionally replicative Ad (CRAd) agents for cancer treatment. One common approach to target virus replication to a specific subset of cells is the use of tissue- or tumor-specific promoters. The COX-2 gene promoter is a specific promoter that has been successfully used to drive CRAd replication in cancer cells [25]. We already demonstrated the COX-2 promoter–driven CRAds suppressed the tumor growth of several gastrointestinal cancers [25,26,27]. On the other hand, to achieve optimal antitumor efficacy of CRAds, we need to develop CRAds with improved tropism to cancer cells. Although the cell attachment of Ad is initiated by the attachment of the fiber protein to the coxsackie adenovirus receptor (CAR), many cancer cells including CRPC lack expression of the CAR, limiting the infectivity of Ads [28,29]. A subsequent step of virus internalization depends on interaction between the Arg-Gly-Asp (RGD) motifs in the Ad5 penton base protein and the integrin molecules on the cell surface [30]. It has been reported that incorporation of an RGD-4C peptide into the HI loop of the fiber knob domain remarkably increased the viral infectivity in CAR-negative cells [31]. Previously, we identified the suitability of an RGD infectivity-enhanced, Cox-2 promoter–driven CRAd for pancreatic cancer [25]. As another strategy to achieve CAR-independent cell entry, fiber-knob chimerism, wherein the fiber knob of Ad5 is replaced with that of the Ad type 3 receptor, has been employed [32]. Our Ad5/Ad3-chimeric, Cox-2 promoter–driven CRAd has also indicated significantly improved oncolysis in esophageal adenocarcinoma [27]. In this context, an infectivity-enhanced Cox-2 promoter–driven CRAd is proposed as a new potential viral therapy agent for mCRPC due to several advantages such as high selectivity, low cytotoxicity, and oncolytic characteristics.

We hypothesize that infectivity-enhanced COX-2 promoter–driven CRAds should be applicable for the development of a novel therapeutic modality for CRPC, especially small-cell prostate cancer and t-NEPC. In this study, we assessed the anticancer effect of Ad fiber modification and the nature of the COX-2 promoter in mCRPC/NEPC cell lines (PC3 and Du-145) and established the functionality of infectivity-enhanced COX-2 CRAds in CRPC/NEPC in vitro and in vivo models.

## 2. Materials and Methods

### 2.1. Cell Lines

The androgen-sensitive (AR-positive) prostate cancer cell line LnCap, and androgen-independent (AR-negative) human prostate cancer cell lines Du-145 (derived from a dural brain metastasis, ATCC HTB-81) and PC3 (derived from a bone metastasis, ACTT CRL-1435) were obtained from American Type Culture Collection (ATCC; Manassas, VA, USA) and maintained as a monolayer in RPMI 1640 (Du-145, LnCap) and Ham’s F12K (PC3) mediums (Mediatech, Herndon, VA, USA). A549 (COX-2-positive lung cancer cell line, ATCC CCL-185) cells and BT474 (COX-2-negative breast cancer cell line, ATCC CRL-3247) cells were cultivated in RPMI 1640. Transformed human embryonic kidney cell line 293 cells (ATCC) were maintained in DMEM (Mediatech). Each medium was supplemented with 10% fetal bovine serum (FBS) (HyClone, Logan, UT, USA), 2 mM L-glutamine, and 100 IU/mL penicillin, and 100 mg/mL streptomycin (Mediatech). The medium for the BT474 was additionally supplemented with bovine insulin (0.01 mg/mL, Life Technologies, Rockville, MD, USA). All cells were incubated at 37 °C in a humidified environment with 5% CO_2_.

### 2.2. Adenoviral Vectors

**Figure 1:**
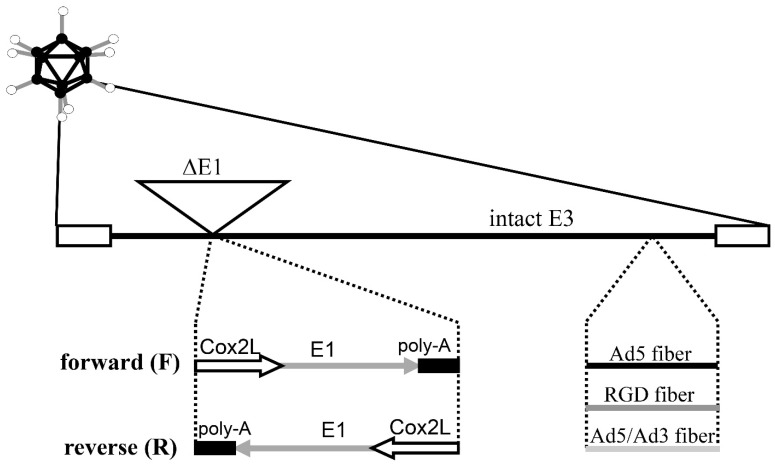
Structure of infectivity-enhanced COX-2 CRAds. All CRAds contain the nt 1-358 sequence of human Ad5, a COX-2 promoter–controlled E1 expression cassette in either forward or reverse orientation (CRAdCox2F and CRAdCox2R), a restored pIX promoter, and an intact E3 region. The fiber was modified either by incorporating an RGD-4C motif into the HI loop of the Ad fiber knob domain (RGD fiber) or by replacing the Ad5 knob with an Ad3 knob (Ad5/Ad3 fiber).

Replication-deficient adenoviral vectors (AdCox2LLuc, AdCox2MLuc, AdCMVLuc) encoding the firefly luciferase (Luc) reporter gene from pGL3-Basic (Promega, Madison, WI, USA) were constructed as previously described [26]. They contain transgene cassettes inserted in place of the deleted E1 region of a common Ad vector backbone. Luc tumor-specific promoter–based AdCox2LLuc and AdCox2MLuc carry the Cox2L (−1432/+59) (long length) and Cox2M (−883/+59) (medium length) promoters derived from phPES2 (provided by Drs. Inoue and Tanabe at the National Cardiovascular Center Research Institute, Japan) [33,34,35]. AdCMVLuc is a control vector that expresses Luc under the ubiquitous cytomegalovirus immediate-early (CMV) promoter [26]. The CMV-promoter-driven luciferase expression vectors with three different fibers (Ad5Luc1, Ad5RGDLuc1, and Ad5/3Luc1) were employed for the assessment of infectivity enhancement. These replication-incompetent Ad vectors have identical structure with the exception of the following modifications. Ad5Luc1 has an unmodified Ad5 fiber [31]. Ad5RGDLuc1 contains an RGD-4C peptide insertion in the HI loop of the Ad5 fiber [31,36]. Ad5/3Luc1 has a chimeric fiber composed of the Ad5 shaft and the Ad3 knob [37]. The genomes of the COX-2 promoter–based CRAds were constructed as described previously [25] (Figure 1). All of them contain a COX-2 promoter–controlled E1 expression cassette in place of the original E1 region of the Ad5 genome. A polyA signal was placed at the end of the E1 expression cassette. There is no deletion or mutation in the E3 region. We constructed two shuttle vectors with the E1 expression cassette in two different orientations: pShuttleCox2LE1-F (5′ to 3′) and pShuttleCox2LE1-R (3′ to 5′) [25]. These shuttle vectors were recombined with Ad5 DNA and pVK503 [36] to generate CRAd genomes containing the wild-type fiber gene and the RGD-modified fiber gene, respectively. To generate COX-2-based CRAds carrying an Ad5/Ad3-chimeric fiber, we used an Ad backbone pMG553 [27], containing the Ad5/Ad3 chimeric fiber sequence. To generate viruses, 293 cells were transfected with PacI-digested plasmids containing the viral genomes. Wild-type Ad5 (AdWt) and its RGD and Ad5/Ad3-chimeric isogenic versions (RGDWt derived from pVK503 and AdMG553 derived from pMG553, respectively) were utilized as nonselective replicative control vectors [37].

The viruses were propagated in E1-transcomplementing 293 cells, purified by double cesium chloride density gradient ultracentrifugation, and dialyzed in phosphate-buffered saline (PBS) with 10% glycerol. Viral aliquots were stored at −80 °C [38]. Titration was performed via a plaque assay and optical-density-based measurement. The viral particles per plaque-forming unit (vp/pfu) ratios for these vectors were in the range of 20 to 80.

### 2.3. Analysis of COX-2 RNA Levels

Total cellular RNA was extracted from semiconfluent cell cultures in 10 cm dishes using the RNeasy mini-RNA extraction kit (Qiagen, Valencia, CA, USA). The COX-2 and the glyceraldehyde-3-phosphate dehydrogenase (GAPDH) RNAs were detected using GeneAmp Gold.

An RNA PCR Core Kit (Perkin-Elmer, Branchburg, NJ, USA) was used as described previously [26]. Briefly, total RNA was reverse-transcribed with oligo-dT primer and murine leukemia virus reverse transcriptase and then amplified by PCR with COX-2- and GAPDH-specific primers. PCR products were analyzed by standard agarose gel electrophoreses.

### 2.4. Promoter and Gene Delivery Analysis with Luciferase-Expressing Ads

These assessments were performed as described previously [25,26]. Prostate cancer and control cells grown in 24-well plates were infected with Ad vectors at a multiplicity of infection (MOI) of 50 pfu/cell for promoter analysis and at 2000 vp/cell for the assessment of fiber modification [27]. The infection medium was replaced with the appropriate growth medium 2 h later. Two days after infection, the cells were lysed with cell culture lysis buffer (Promega), and Luc activity was determined with the Luciferase Assay System (Promega). Experiments were performed in triplicate and standardized with protein concentrations quantified by the DC protein assay (Bio-Rad, Hercules, CA, USA).

### 2.5. In Vitro Analysis of Cytocidal Effect by Crystal Violet Staining

To analyze virus-mediated cell killing, 25,000 cells/well were plated in 12-well plates and infected with the viruses in 200 µL of infection medium containing 5% FBS at either 0.01 or 0.1 vp/cell [25]. After 3 h, 1 mL of the growth medium was added. Two days later, the infection medium was replaced with 1% FBS medium. After 12 days (8 days for the LnCap cell line) of cultivation, the cells were fixed with 10% buffered formalin for 10 min and stained with 1% crystal violet in 70% ethanol for 20 min, followed by washing with water and drying.

### 2.6. Quantitative In Vitro Cytotoxicity Assay

Cells (3000/well) cultured in 96-well plates were infected with Ad vectors at 1.0 vp/cell in 100 µL of 5% FBS medium. On the next day, 100 µL of the growth medium containing 1% FBS was added. The cells were incubated for 18 days (14 days for LnCap), and the number of surviving cells was analyzed by a colorimetric method using the Cell Titer 96 Aqueous Nonradioactive Cell Proliferation Assay (Promega) as described by the manufacturer. Absorbance was measured at a wavelength of 490 nm in an E-Max spectrophotometer 11 (Molecular Device Corporation, Sunnyvale, CA, USA), and standard curves were generated by analyzing the known number of live cells [27]. Based on this curve, the number of living cells was calculated for the experimental groups using SoftMax computer software (Molecular Device Corporation). All experiments were repeated in triplicate.

### 2.7. In Vivo Antitumor Effect in a Prostate Cancer Xenograft Model

Female ncr/nu nude mice (Frederick Cancer Research, Frederick, MD, USA) (6–8 weeks of age) were used to establish prostate cancer xenografts. Du-145 cells (3.2 × 10^6^ per injection site) or PC3 cells (2.8 × 10^6^ per injection site) were inoculated into the flanks of the mice. When the nodules reached a size of 6–8 mm in maximum diameter, a single virus dose (5 × 10^9^ vp in 100 µL PBS) of CRAd or control viruses was injected intratumorally. The condition of the mice was monitored daily, and the tumor diameter was measured twice per week with calipers. The tumor volume was calculated using the formula: tumor volume = (width^2^ × length)/2. In accordance with institutionally approved animal experimental protocol, the mice were euthanized 65 (Du-145) or 90 (PC3) days after viral injection. All animals received humane care based on the guidelines set by the American Veterinary Association. All the experimental protocols involving live animals were reviewed and approved by the Institutional Animal Care and Use Committee of the University of Alabama at Birmingham.

### 2.8. Statistical Methods

Statistical analysis was performed with a two-tailed *t*-test. Data are expressed as the mean ± standard deviation of at least three sets of results. Results were considered statistically significant when *p* < 0.05.

## 3. Results

### 3.1. Transcriptional Status of COX-2 in Prostate Carcinoma Cell Lines

**Figure 2:**
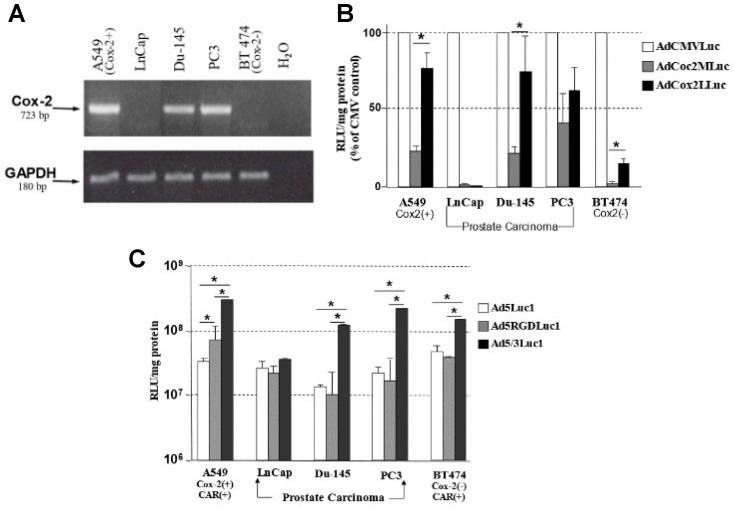
In vitro analyses. (**A**) Analysis of COX-2 RNA levels in prostate cancer cell lines. Total cellular RNA of human prostate cancer cells (LnCap, Du-145, PC3) as well as COX-2 control cells (A549 as positive and BT474 as negative) was reverse-transcribed with oligo-dT primer and amplified by PCR with COX-2—(**upper** panel) and GAPDH (**lower** panel)-specific primers. The signal for correctly spliced cyclooxygenase-2 mRNA was detected as a 723 bp band. (**B**) Activity and selectivity of COX-2 promoter in prostate cancer cells. Human prostate cancer cells (LnCap, Du-145, PC3), COX-2—positive A549 cells, and COX-2-negative BT474 cells were infected with AdCMVLuc, AdCox2MLuc, and AdCox2LLuc. The cells were lysed and assessed for luciferase activity two days after infection. Data are shown as percentages of relative light units (RLUs) per milligram protein in relation to that of CMV-promoter-driven luciferase expression. The Cox2M promoter showed higher activity in Du-145 cells compared to the Cox2L promoter, and both had about the same activity in PC3. Minimal activity was seen in the COX-2-negative prostate cancer cell line (LnCap) and the COX-2-negative control cell line (BT474). Errors bars represent the standard deviation calculated from three replicates. * *p* < 0.05 (**C**) Transduction efficiency in prostate cancer cells with infectivity-enhanced Ad vectors. Human prostate cancer cells (LnCap, Du-145, PC3), and two control cell lines (A549 and BT474) were infected with CMV-promoter-driven Luc-expressing vectors possessing unmodified, RGD-modified, or Ad5/Ad-3chimeric fibers (Ad5Luc1, Ad5RGDLuc1, and Ad5/3Luc1, respectively). After two days of incubation, the cells were lysed, and the Luc activity was measured. Du-145 and PC3 prostate cancer cell lines demonstrated significantly higher levels of transgene expression with the Ad5/Ad3—modified vector than that with Ad5Luc1. The enhancement was not significant for LnCap. In all three prostate cancer cell lines, the level of transgene expression with an RGD-modified vector tended to be slightly lower than that of Ad5Luc. Errors bars represent the standard deviation calculated from three replicates. * *p* < 0.05.

The COX-2 mRNA status of the cell lines used for this experiment was analyzed by reverse-transcription (RT)-PCR (Figure 2A). As a COX-2-positive cell line, we used A549 (lung cancer cell line) that was previously confirmed (26, 27). Both mCRPC cell lines (Du-145, PC3) showed high levels of COX-2 mRNA comparable to that of the COX-2-positive control (A549 lung cancer). LnCap (androgen-sensitive prostate cancer cell line) and BT474 (breast cancer COX-2-negative control cell line) were negative for COX-2 mRNA. GAPDH mRNA levels were the similar among all the cells tested, serving as a control for the RNA isolation and detection procedure.

### 3.2. The Selectivity of the COX-2 Promoter for Prostate Cancer

To analyze the activity of the COX-2 promoter in the Ad vector, two Luc expression vectors with two different lengths of the COX-2 5′upstream control region (Cox2M and Cox2L) were tested in prostate cancer cells (Figure 2B). The Cox2L promoter showed significantly higher activity in Du-145 cells compared to the Cox2M promoter, while both were about the same in PC3. However, even in COX-2-expressing prostate cancer cell lines, the COX-2 promoter was weaker than the CMV promoter that has been used as an extremely strong promoter. In contrast, the COX-2 promoter–driven luciferase expression vector showed only background-level activity in the COX-2-negative androgen-sensitive prostate cancer cell line (LnCap) and the COX-2-negative control cells (BT474). These data indicate that the COX-2 promoter maintained the designed selectivity in prostate cancer cells after configuration into the vector.

### 3.3. Analysis of Transduction Efficiency of RGD- and 5/3-Modified Vectors in Prostate Cancer

To assess the transduction efficiency and determine whether the incorporation of an RGD-4C motif into the HI loop of the Ad fiber knob domain or replacement of the Ad5 knob with the Ad3 knob would enhance the infectivity of Ad vectors in prostate cancer cells, three replication-deficient CMV-promoter-driven Luc expression vectors with unmodified (Ad5Luc1), RGD-modified (Ad5RGDLuc1), and Ad5/Ad3-chimeric (Ad5/3Luc1) fibers were tested in prostate cancer cell lines (Figure 2C). Du-145 and PC3 prostate cancer cell lines demonstrated significantly higher levels of transgene expression with the Ad5/Ad3-modified vector than that with Ad5Luc1, reflecting the resistance of these cells to infection by an Ad vector with wild-type Ad5 fiber. The enhancement was not significant for LnCap, since this cell line can be easily infected with an Ad vector without fiber modification. In all three prostate cancer cell lines examined, the level of transgene expression with the RGD-modified vector did not show significant difference compared to the one with the unmodified fiber.

### 3.4. Increased Oncolytic Efficiency of Infectivity-Enhanced, COX-2 CRAds In Vitro

**Figure 3:**
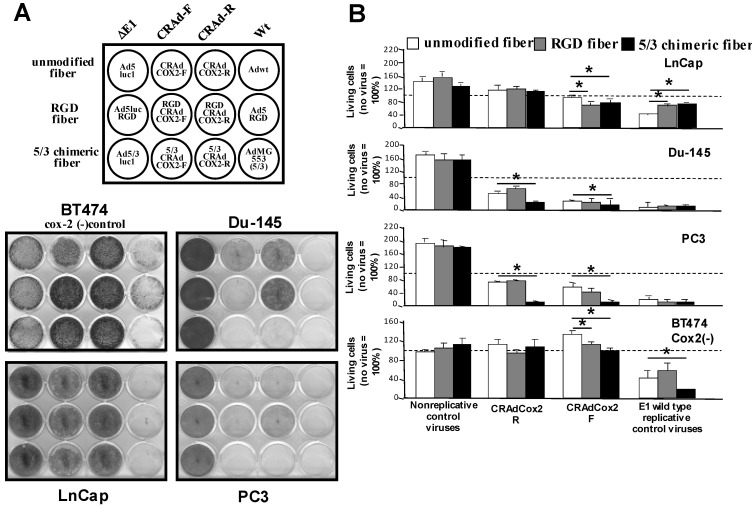
In vitro cytocidal effect of infectivity-enhanced, COX-2 CRAds in prostate cancer cell lines. (**A**) Crystal violet assay. Human prostate cancer cells (LnCap, Du-145, PC3), and COX-2 control cells (A549 and BT 474) were infected with CRAds at an MOI of 1 vp/cell. After 12 (Du-145, PC3) or 8 days (LnCap), the adenoviral cytotoxicity was analyzed by crystal violet staining. In prostate cancer cell lines, COX-2 CRAd showed a significant cytocidal effect in COX-2-positive Du-145 and PC3 cell lines, while it showed no significant effect on BT474 (COX-2-negative control cells) and LnCap (COX-2-negative) prostate cancer cells. CRAds with the left to right-direction E1 expression cassette (CRAdCox2F, RGDCRAdCox2F, 5/3CRAdCox2F) exhibited stronger oncolytic effect than CRAds with the reverse-direction cassette (CRAdCox2R, RGDCRAdCox2R, 5/3CRAdCox2R). The cytotoxicity of CRAds with different fibers was similar to that of the corresponding replicative control viruses containing the wild-type early genes (Ad5Wt, RGDWt, AdMG553). The nonreplicative controls (Ad5Luc1, RGDAd5Luc1, 5/3Ad5Luc1) did not cause any oncolysis. Data are representative samples from at least three experiments performed on each cell line. (**B**) Quantitative in vitro cytotoxicity assay. Human prostate cancer cells (LnCap, Du-145, PC3), COX-2-positive A549 cells, and COX-2-negative BT474 cells were infected with Ad vectors at 1.0 vp/cell. The cells were incubated for 18 days (14 days for LnCap), and the number of surviving cells was analyzed by a colorimetric method. All COX-2-based CRAds demonstrated oncolytic killing in CRPC cells (Du-145 and PC3) while no cytocidal effect was observed in the COX-2-negative control (BT474) and the COX-2-negative prostate cancer cell line (LnCap). In the Du-145 cell line, CRAdCox2R with the 5/3 modification showed an augmented cytocidal effect compared to fiber-unmodified and RGD-modified counterparts. In the PC3 cell line, CRAds with the 5/3 modification (F and R) were stronger than their fiber-unmodified and RGD-modified counterparts. * *p* < 0.05.

The concepts of COX-2 promoter transcriptional targeting and Ad tropism modification were configured into a replicative virus. We assessed the selective oncolytic effect of COX-2 CRAds in prostate cancer cells lines and in COX-2-negative control cell line BT474. In prostate cancer cell lines, COX-2 CRAds showed a significant cytocidal effect in COX-2-positive mCRPC cell lines (Du-145 and PC3) while there was no significant effect on LnCap (COX-2-negative prostate cancer cells) and BT474 (COX-2-negative control cells) (Figure 3A). CRAds with the left to right–direction E1 expression cassette (CRAdCox2F, RGDCRAdCox2F, 5/3CRAdCox2F) exhibited a stronger oncolytic effect than those with the reverse direction cassette (CRAdCox2R, RGDCRAdCox2R, 5/3CRAdCox2R). The cytocidal effect of Ad5/Ad3 CRAds in COX-2-positive mCRPC cells was similar to that of the replicative control viruses containing the wild-type E1 genes (Ad5Wt, RGDWt, AdMG553). Of note, all COX-2 CRAds maintained their replication specificity after fiber modification because none of them affected the COX-2-negative control’s (BT474) viability. The nonreplicative controls (Ad5Luc1, Ad5RGDLuc1, Ad5/3Luc1) did not induce any oncolysis. The replication and cytocidal effects of CRAds were further confirmed with a quantitative cell viability assay (Figure 3B). In COX-2-expressing mCRPC cell lines (Du-145 and PC3), all COX-2-based CRAds, especially 5/3CRAdCox2, showed strong oncolysis. However, no cytocidal effect was observed in the COX-2-negative prostate cancer cell line (LnCap) and the COX-2-negative control (BT474).

### 3.5. Therapeutic Efficacy of Infectivity-Enhanced CRAds In Vivo

**Figure 4:**
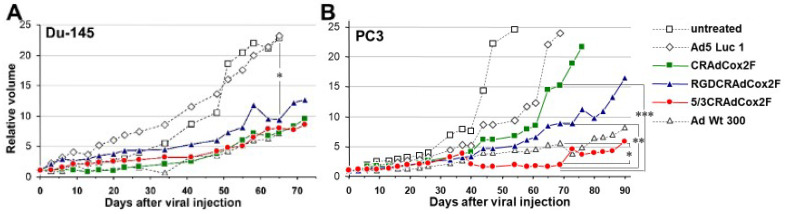
In vivo antitumor effect in subcutaneous xenograft model. The in vivo antitumor effect of COX-2 CRAds was analyzed in Du-145 (**A**) and PC3 (**B**) hormone-refractory prostate cancer cell lines. Subcutaneous xenografts in nude mice were treated with a single intratumoral injection of 5 × 10^9^ vp of each virus in 100 µL PBS. The tumor volume was shown as the relative volume compared to day 0. In Du-145, both the untreated group and the group with nonreplicative control virus had to be terminated due to the size of the tumors on day 65, while the control groups were sacrificed on day 54 and 69, respectively. For the Du-145 xenograft, the CRAd Cox2F, RGDCRAdCox2F, 5/3CRAdCox2F, and AdWt groups started to show significant tumor suppression compared to the nonreplicative control group starting on day 65. In PC3 xenografts, CRAdCox2F and RGD CRAd Cox2F showed a slight antitumor effect, but it was not significant when compared to the untreated or nonreplicative control groups. The size of the tumors treated with chimeric 5/3CRAdCox2F and AdWt was significantly reduced (*p* < 0.05) compared to that in the nonreplicative control group from day 44. The group with 5/3CRAdCox2F showed significant tumor suppression (*p* < 0.05) compared to the group with CRAdCox2F or RGD CRAd Cox2F from day 40 to the end of the experiment. At day 69, the relative tumor volume of the 5/3CRAdCox2F group was significantly smaller than that of the groups with CRAdCox2F, RGDCRAdCox2F, and AdWt. * *p* < 0.05, ** *p* < 0.01, *** *p* < 0.005.

The in vivo analysis of antitumor efficacy was performed using a subcutaneous xenograft model in nude mice. Established tumors of Du-145 and PC3 were treated with a single intratumoral injection of 5 × 10^9^ vp of each virus, and the tumor size was monitored (Figure 4 and Supplementary Figure S1).

In Du-145 xenografts (Figure 4A), CRAd Cox2F and 5/3CRAdCox2F started to show significant tumor suppression compared to nonreplicative control as early as day 9, followed by RGDCRAdCox2F from day 23. When the antitumor effect was compared to the untreated control, the significance appeared with a little delay. At the end of the experiment (day 65), all four replication-competent viruses showed a statistically significant antitumor effect. Compared to the nonreplicative Luc expression vector (relative tumor volume 23.14 ± 12.9, *n* = 8), CRAd Cox2F (relative tumor volume 9.39 ± 6.46, *n* = 7, *p* = 0.033), RGDCRAdCox2F (relative tumor volume 9.41 ± 3.51, *n* = 8, *p* = 0.030), 5/3CRAdCox2F (relative tumor volume 8.06 ± 4.71, *n* = 9, *p* = 0.021), and AdWt (relative tumor volume 7.13 ± 2.8, *n* = 4, *p* = 0.016) groups showed significant tumor growth suppression on day 65.

In PC3 xenografts, the benefit of fiber modification was more significant (Figure 4B). The CRAdCox2F (*n* = 9) and RGD CRAd Cox2F (*n* = 8) groups showed a marginal antitumor effect against the untreated (*n* = 7) or nonreplicative Ad5Luc1 groups (*n* = 6) throughout the time course. In contrast, starting at day 44, the size of the tumors treated with chimeric 5/3CRAdCox2F (*n* = 8) was significantly reduced (*p* < 0.05) compared to those with nonreplicative control. Of note, compared to the group receiving CRAdCox2F or RGD CRAd Cox2F, the 5/3CRAdCox2F group showed significant tumor suppression (*p* < 0.05) from day 40 until the end of the experiment. Most importantly, compared to the replication-competent wild-type Ad (AdWt), 5/3CRAdCox2F showed a more potent antitumor effect between day 40 and day 76 (*p* < 0.05). The most significant difference was observed at day 69: the relative tumor volume of the 5/3CRAdCox2F group (1.8 ± 0.5, *n* = 8) was significantly smaller than that of the groups with CRAdCox2F (15.2 ± 8.7, *n* = 9, *p* = 0.0008), RGDCRAdCox2F (8.8 ± 3.3, *n* = 8, *p* = 0.0005), and AdWt (5.6 ± 3.1, *n* = 6, *p* = 0.022). Compared to the marginal augmentation provided by fiber modification in the Du-145 cell line, 5/3Cox2CRAdF showed a dramatically stronger in vivo antitumor effect over those with unmodified or RGD fibers in the PC3 mouse xenograft model.

## 4. Discussion

Prostate cancer has many treatment options such as radical prostatectomy, radiotherapy, or brachytherapy as long as it is confined within the prostate. However, in patients with recurrent or advanced disease, especially mCRPC, treatment remains palliative. Before 2010, docetaxel chemotherapy was the only treatment showing a survival advantage, which is reflected in its approval by the U.S. Food and Drug Administration and in its widespread use as the first-line therapy globally [39,40]. More recently, several large, randomized clinical trials have led to the approval of new agents for the treatment mCRPC. New therapies have all demonstrated an overall survival benefit in mCRPC patients [41]. Although these new agents can provide improved prognosis in mCRPC patients [42], development of mCRPC must make it difficult to control the PC progression and the metastasis-related symptoms, and thus the treatment for mCRPC remains very challenging. Furthermore, patients with small-cell prostate cancer or those showing neuroendocrine transdifferentiation after hormonal treatment (treatment-related neuroendocrine prostate cancer, t-NEPC) show strong resistance to multiple therapies and represent an unmet medical need [8,9]. Here, we demonstrated that infectivity-enhanced, COX-2 promoter–driven CRAds showed an anticancer effect in mCRPC/t-NEPC cells and should be a promising option in a novel therapeutic strategy for mCRPC/NEPC patients.

The promoters responsible for the expression profile of PC serum markers (e.g., PSA, PSMA) have been used for targeting PC by virtue of the contrast observed in patients [43,44]. However, the expression of most PC markers depends on androgen signaling, and the aforementioned promoters have androgen-responsive elements (AREs) as a core promoter region [45,46]. While AR gene amplification and its overexpression are observed in about 30% of PC samples [47], approximately 20% of PC does not show PSA elevation [48]. In addition, AR-null or AR-low cells and tissues are commonly observed [47,48,49,50]. In this sense, ARE-dependent promoters might not be always suitable for therapeutic targeting of CRPC. On the other hand, COX-2 is overexpressed in a variety of cancers including PC [51], and this promoter does not include AREs [33,34,35], such that the activity will not be affected by the intracellular androgen signaling status. Moreover, COX-2 was reported to have crucial roles in cancer progression in androgen-independent PC [19]. The results in this study showed that the mCRPC cell lines, Du-145 and PC3 (which are derived from dural and bone metastasis, respectively) exhibited a higher expression of COX-2. Thus, employing the COX-2 promoter is reasonable way for promoter-driven virotherapy for mCRPC. Prostate-specific promoter–based oncolytic adenovirus vectors have previously been established, and some of them exhibited antitumor effect in PC cells [52]. However, the oncolytic effect of COX-2 promoter–driven CRAds has not been reported in mCRPC. In the PC3 and Du-145 cell lines, the luciferase expression vector with the long COX-2 promoter (≅1.5 kb) yielded very strong luciferase activity comparable to the CMV promoter. However, the activity in LnCap cells was as low as that in COX-2-negative control cells. These results closely complied with the COX-2 RNA status assessed by RT-PCR assay. In this sense, the COX-2 promoter maintained tight selectivity in the adenoviral vector structure. Since the role of COX-2 in PC progression is well established [15,53], application of this promoter for PC, especially CRPC, in the gene therapy context is promising.

Although adenoviral vectors show relatively high infectivity in a variety of cancers, its clinical application has been compromised in some cancers due to poor transduction. In our studies, CRPC cells share the same low infectivity as fiber-unmodified Ad due to the paucity of CAR expression. To overcome this barrier, a CAR-independent vector system is desperately needed for CRPC. To assess the transduction efficiency with different fibers, we used three identical, replication-deficient, CMV-promoter-driven Luc expression vectors with wild-type (Ad5Luc1), RGD-modified (Ad5RGDLuc1), and Ad5/Ad3-chimeric (Ad5/3Luc1) fibers (Figure 2C). When the vectors with an RGD-4C motif incorporated into the HI loop (AdRGDLuc1) and a replacement of the Ad5 knob region with the Ad3 knob (Ad5/3Luc1) were compared to fiber-unmodified counterpart (Ad5Luc1) in PC3 and Du-145 cell lines, the Ad5/Ad3 chimera demonstrated significantly higher levels of transduction in vitro while the RGD-modified vector did not show enhancement. In fact, CAR expression of Du-145 is limited and that of PC3 is close to zero according to flow cytometry. The enhancement in LnCap and A549 was minimal because vectors with a normal fiber infected these CAR-positive cells efficiently. Based on these results, we constructed fiber-modified COX-2 CRAds and tested them in CRPC cell lines. When the cytocidal effect of CRAds were assessed by crystal violet staining, all COX-2 CRAds showed replication and subsequent oncolysis in the COX-2-positive CRPC cell lines, while the COX-2-negative LnCap cell line was not affected. The Ad5/Ad3 chimera demonstrated the strongest cytocidal effect among all COX-2 CRAds in both PC3 and Du-145 cell lines. The analysis performed with the MTS assay quantitatively confirmed the same tendency. These data indicate that the infectivity-enhanced COX-2 CRAds are promising therapeutic agents for CRPC. As the next step, in vivo antitumor efficacy was assessed in Du-145 and PC3 CRPC subcutaneous xenograft models in nude mice. In Du-145 xenografts, all three COX-2 CRAds with different fibers exhibited a significant antitumor effect compared to the untreated and nonreplicative controls, and the effect was comparable to the positive control with wild-type Ad. However, in Du-145, the effect was similar among the three CRAds with different fibers and was comparable to the positive control with wild-type Ad5. In the case of PC3, tumor suppression from COX-2 CRAds with unmodified and RGD fibers was not significant. In contrast, the antitumor effect from 5/3 COX-2 CRAd was significantly greater than that of all other groups. It is noteworthy that the 5/3 COX-2 CRAd was even stronger than the positive control (fully replicative) wild-type Ad5 for the period from day 40 to 69. Compared to Du-145, CAR expression in PC3 is extremely low (PC3: 1.7%, Du-145: 22.8%). This may explain why the augmentation of the antitumor effect with the 5/3 modification was more evident in PC3 than in Du-145.

In this study, we evaluated the oncolytic effect of the infectivity-enhanced COX-2 CRAds in NE-like PC cell lines Du-145 and PC3 from brain and bone metastases [8,11,12,54]. The model we used in our study represents one of the most refractory group in PC patients because NE-like PC arising after hormonal therapy shows poor prognosis due to aggressive clinical course and unresponsiveness to hormonal therapies [55]. Interestingly, overexpression of COX-2 was reported to be associated with the metastatic progression of PC cells with an acquired NEPC phenotype [56]. With respect to the mechanism of the cancer-selective effect of COX-2 CRAds, it has been reported that the function of control elements in the COX-2 promoter region plays an important role and that the cyclic-AMP-responsive element is particularly important for the constitutive induction of COX-2 expression in cancer cells [57]. In addition, increasing evidence supports the role of COX-2 in cancer progression, such as promoting tumor cell proliferation, epithelial-to-mesenchymal transition, and cancer invasion [58,59,60]. Therefore, our infectivity-enhanced COX-2 CRAds might show a favorable effect for NEPC as well, and further works are required to elucidate the efficacy of infectivity-enhanced COX-2 CRAds in the more specific target of mCRPC. It is important to have various modalities in a clinician’s armamentarium since cancers we encounter in the clinical settings are molecularly and phenotypically diverse. There are several fiber-modified CRAds currently in the preclinical stage. The elucidation of in vivo features and toxicity in humans in these projects will provide valuable data for the understanding of CRAd behavior in the human body. In the context of COX-2 CRAd application in NEPC, we believe the left-to-right direction (F) is more promising because it shows stronger cytocidal effects in Figure 3A. However, if the toxicity in the preclinical toxicological study is potentially problematic, we can still use an R-direction virus that has less leakiness of COX-2-promoter activity in COX-2-negative cells. We hope that such clinical translation efforts will lead us to develop an upcoming clinically functional virotherapy modality for CRPC in the near future.

In conclusion, we demonstrated that 5/3-modified COX-2 CRAds showed a potent anticancer effect in mCRPC cells in vitro and in vivo. The infectivity-enhanced COX-2 CRAd is a potentially promising therapeutic agent for the treatment of mCRPC/NEPC, and the standard therapeutic strategy combined with this virus may provide better disease control and improvement of prognosis in such advanced PC patients with very few alternative therapies.

## Data Availability

Further information on the presented data as well as the raw data is available on request.

## Supplementary Materials

The following supporting information can be downloaded at: https://www.mdpi.com/article/10.3390/v15040901/s1, Figure S1: In vivo antitumor effect in subcutaneous xenograft model.
