# Supplementary material for: Infectivity-Enhanced, Conditionally Replicative Adenovirus for COX-2-Expressing Castration-Resistant Prostate Cancer
Source: Viruses. 2023 Mar 31;15(4):901. doi: 10.3390/v15040901 (PMC10144787; doi:10.3390/v15040901)
Supplement: Supplementary file 1 [file viruses-15-00901-s001.zip › viruses-2228696-supplementary.pdf]

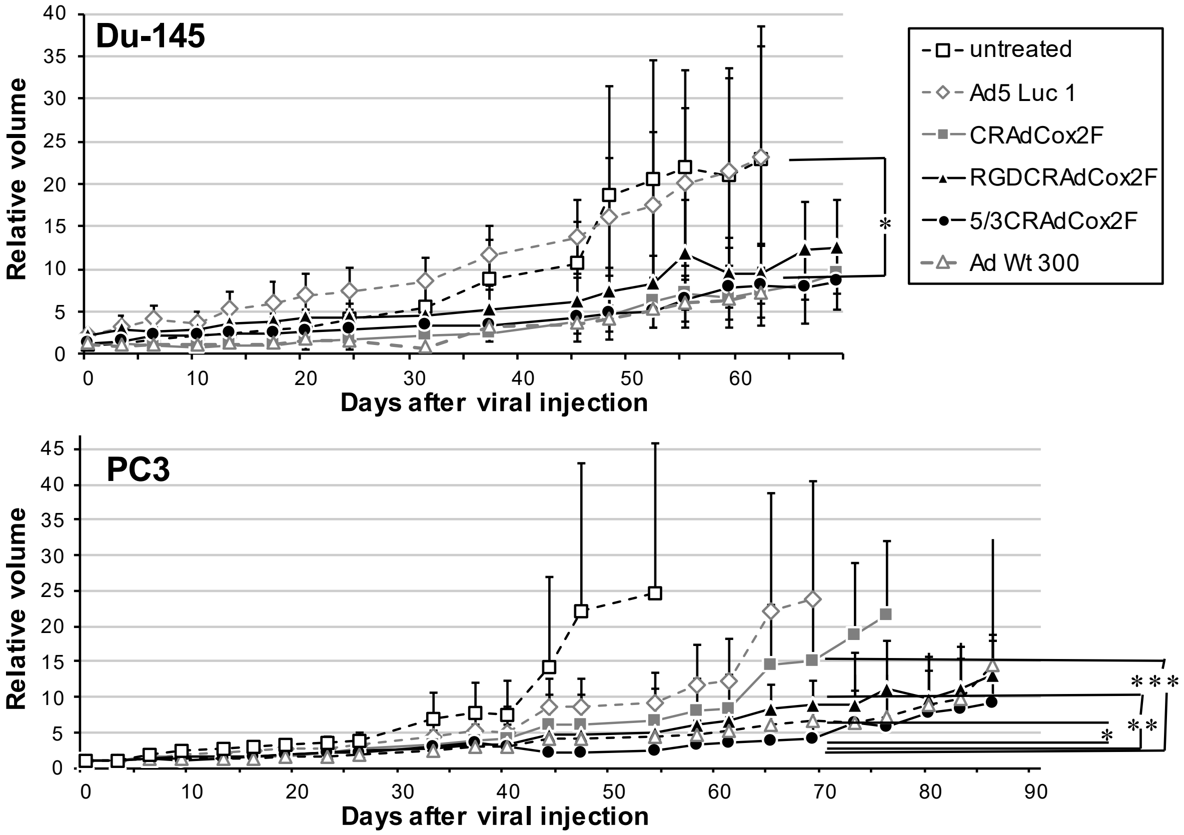

Supplementary figure S1. In vivo antitumor effect in subcutaneous xenograft model.
The in vivo antitumor effect of COX-2 CRAds was analyzed in Du145 and PC-3 hormone-refractory prostate cancer cell lines. The error bars indicate standard deviation. *p<0.05, **p<0.01, ***p<0.005
